# Accurate non-invasive image-based cytotoxicity assays for cultured cells

**DOI:** 10.1186/1472-6750-10-43

**Published:** 2010-06-17

**Authors:** Patricia Marqués-Gallego, Hans den Dulk, Claude Backendorf, Jaap Brouwer, Jan Reedijk, Julian F Burke

**Affiliations:** 1 Leiden Institute of Chemistry, Gorlaeus Laboratories, Leiden University, PO Box 9502, 2300 RA Leiden, The Netherlands; 2 Genetix Limited, Queensway, New Milton, Hampshire, BH25 5NN, UK

## Abstract

**Background:**

The CloneSelect™ Imager system is an image-based visualisation system for cell growth assessment. Traditionally cell proliferation is measured with the colorimetric MTT assay.

**Results:**

Here we show that both the CloneSelect Imager and the MTT approach result in comparable EC_50 _values when assaying the cytotoxicity of cisplatin and oxaliplatin on various cell lines. However, the image-based technique was found non-invasive, considerably quicker and more accurate than the MTT assay.

**Conclusions:**

This new image-based technique has the potential to replace the cumbersome MTT assay when fast, unbiased and high-throughput cytotoxicity assays are requested.

## Background

High-throughput screening methods for toxicology evaluation in the early phases of drug discovery are highly desired. In the early 1980´s a novel assay for cell survival determination was reported [1], which has been frequently used to study the biological activity of a variety of potential cytostatic drugs. The assay was presented as a rapid, precise and simple method to detect living cells in mammalian cell cultures, using the tetrazolium salt MTT (3-(4,5-dimethylthiazol-2-yl)-2,5-diphenyl tetrazolium bromide) and a microtiter plate reader. The MTT is reduced in living cells, forming formazan crystals which are then dissolved in a mixture of HCl and isopropanol [1], or alternatively in dimethylsulfoxide [2]. The optical density of dissolved crystals is then read in each well within the first hour. Cell survival is determined after the data analysis [1]. Although widely used, the MTT assay has several disadvantages: in particular poor linearity with the cell number, sensitivity to environmental conditions and more importantly the dependence of cell metabolism on formazan [3-5]. These observations have lead to a search for different alternative assays, such as the measurement of cellular protein content with the SRB dye (Sulforhodamine B) [4,6,7] and a variety of other *in vitro *assays [8]. However, the SRB assay requires a time-consuming workout, such as incubation with the dye for 30 min and fixation. A faster and more reliable system able to screen large number of potential compounds in a large panel of cells would therefore be considered an enormous improvement in the pharmacology research. The CloneSelect™ Imager (Genetix Limited, New Milton,[9]) could offer such an opportunity. The system was designed to be capable of screening cellular confluence and growth in a range of microwell plates. For these measurements, the first step consists on a calibration of the cell number in the microtiter plates in used, and for each cell type. After this step, the cells will be counted based on such calibration. Cell growth is viewed and tracked in every well, using label-free white light imaging for consistent determination of cell confluence and cell number estimation. Therefore, from such measurements the confluence in each well can be determined rapidly and efficiently. The CloneSelect Imager system captures images of the entire well, which provides a label-free, non-destructive and direct analysis of the cell proliferation in each well as function of time. Moreover, the imaging of a 96 well plate is extremely fast, with the data acquisition in less than 3 min. Data can be either output into a spread sheet, or displayed visually as a series of pie charts.

The present study attempts to validate the CloneSelect™ Imager system for use in cellular toxicity screening of platinum-based drugs. Two different drugs have been selected for this study, namely the well-known cisplatin and oxaliplatin in two different human cancer cell lines. A comparison is made between the MTT assay results and the results obtained using the CloneSelect Imager system using a Bland-Altman plot [10].

Our results unequivocally show that the CloneSelect™ Imager system offers a superior and rapid alternative in determining the cytotoxicity of different agents on both adherent and non-adherent cell lines.

## Results and discussion

**Figure 1:**
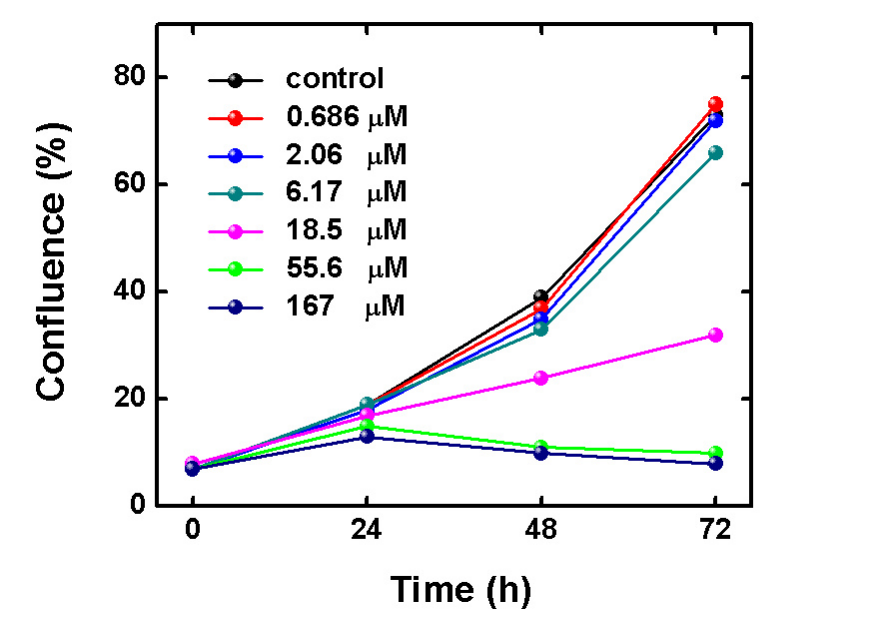
**Cell confluence (%) *vs *incubation time with cisplatin in the A2780R cells; only results of six wells are plotted for clarity**.

Plates were treated with the antiproliferative agents, imaged with the CloneSelect Imager system 24 h, 48 h and 72 h after the addition of the agents. To compare with the MTT assay, once the plate was imaged with the CloneSelect Imager system, the MTT assay was performed; therefore, the results from the CloneSelect™ Imager and the MTT assay originate from the same plate and therefore are directly comparable. The well-known human ovarian carcinoma cisplatin-sensitive A2780 cells and the cisplatin-resistant counterpart A2780R cells were selected as adherent cells. The cytotoxic activity of cisplatin and oxaliplatin has been investigated in parallel cultures under the same conditions in a pair of human ovarian carcinoma cell lines (i.e. the A2780 and the A2780R cells). The incubation with the platinum(II) compounds can be followed using the CloneSelect Imager system, obtaining a rapid view of the antiproliferative activity of the compounds in the cells over the time (See Additional file 1, Figure S1). The cell confluence (%) in the wells *versus *time of incubation (h) with the cisplatin in the A2780R cells is shown in Figure 1.

The compound was added to the cells in different concentrations to achieve a dose-response curve. The A2780R cells were still growing 24 h after the addition of the compounds (Figure 1); therefore, the determination of the EC_50 _value was not performed in this case. This observation is important, since the EC_50 _value after 24 h of incubation with cisplatin in the A2780 cell line has been reported [11-13]. The same plate after 48 h of incubation with the platinum(II) compound shows significant changes. The cells incubated with higher concentrations show a lack of growth, while the cells incubated with lower concentrations show a growth comparable to the non-treated cells. The growth inhibition of the cells is even more evident in the wells with highest concentration of the platinum compound when the same plate is imaged after 72 h of incubation, while the growth continue in the wells with lower concentrations of the antiproliferative agent. Similar results were obtained for cisplatin incubation in the A2780 cells (See Additional file 2, Figure S2), as also observed for the incubation with oxaliplatin (data not shown).

**Figure 2:**
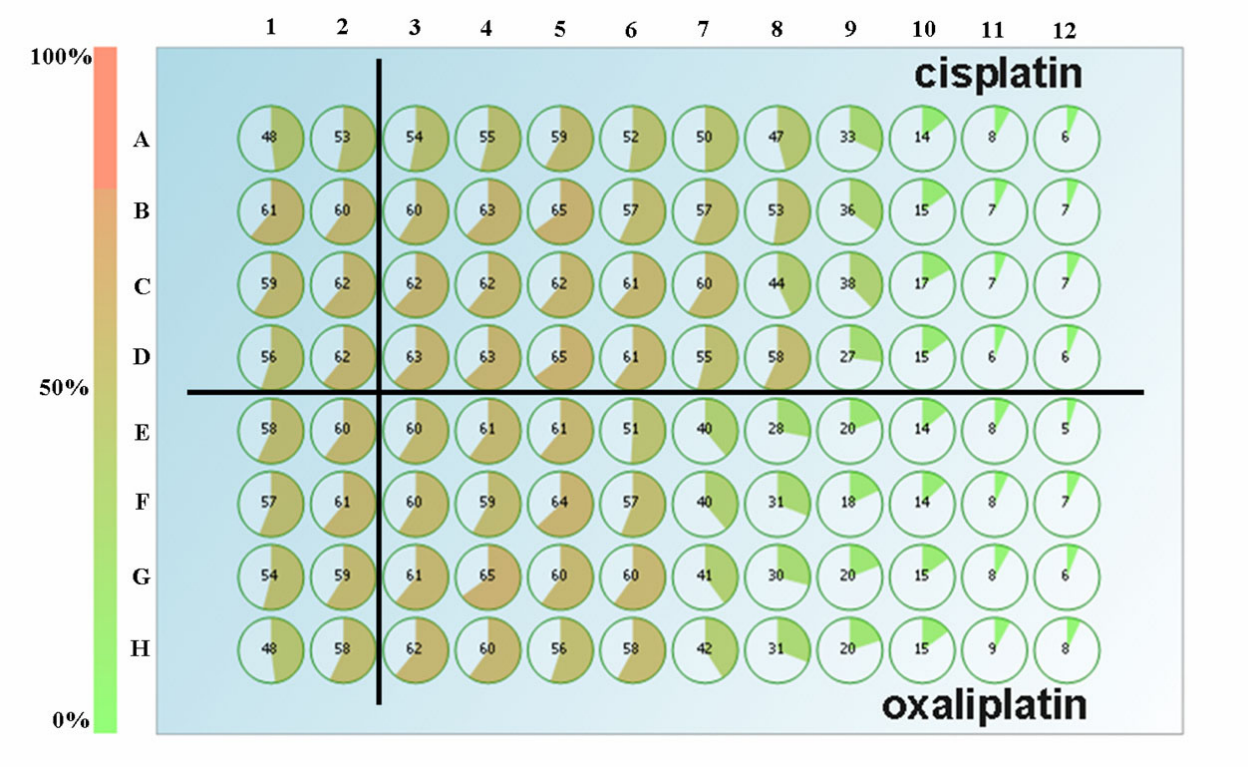
**Cell confluence (in %) of the A2780R cells treated with cisplatin (rows from A to D), or oxaliplatin (rows from E to H) after 72 h of incubation**. First two columns (1 and 2) are non-treated cells. The compounds concentration is increasing from well 3 to well 12 (from the left to the right: 8.47 nM, 25.4 nM, 76.2 nM, 0.229 μM, 0.686 μM, 2.06 μM, 6.17 μM, 18.5 μM, 55.6 μM, and 167 μM).

Cell confluence (%) is also displayed directly with the CloneSelect Imager system, as depicted in Figure 2, where the two first columns (1 and 2) of the microtiter plate were non-treated cells. In addition, the plate was divided to incubate the A2780R cells with both cisplatin and oxaliplatin, as described in the experimental section, using ten different concentrations. Figure 2 clearly shows that the CloneSelect Imager system provides a rapid insight into the different cytotoxic activities displayed by both platinum drugs against the cells under study. The A2780R cells were treated with the same concentrations of cisplatin and oxaliplatin, confirming that oxaliplatin is more cytotoxic than cisplatin in the A2780R cells. Similar results as for the cytotoxicity of oxaliplatin in the A2780R cells were observed in the A2780 cells (see Additional file 3: Figure S3).

Moreover, it is important to mention that a different sensitivity of the A2780R cells against cisplatin was observed, compared to the sensitivity of the A2780 cells. For instance, in Figure 2 the non-treated A2780R cells have a 55% confluence, while a 33% of confluence is observed when the cells are incubated with 6.17 μM of cisplatin. In the case of A2780 cells (see Figure S3) the sensitivity to the drug is higher, finding a 15% of confluence after 72 h of incubation with 6.17 μM cisplatin (non-treated cells 40%).

### Comparison of cytotoxicity determined by CloneSelect™ Imager system and the MTT assay

**Figure 3:**
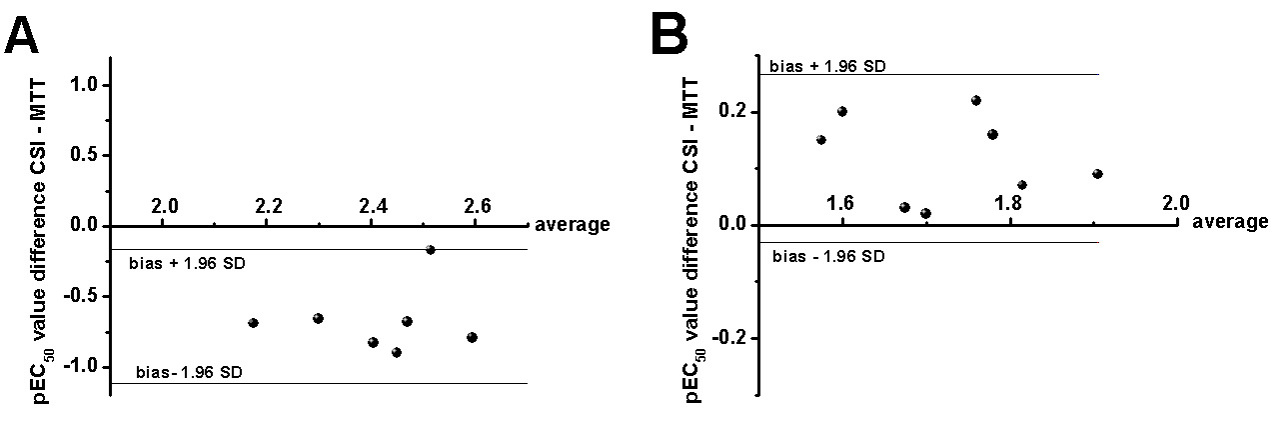
**Bland-Altman plot (pEC_50 _value difference CloneSelect Imager-MTT *vs *average) showing the comparison of the pEC_50 _values produced by cisplatin in: (A) the A2780 cells, (B) the A2780R cells, after 48 h of incubation, tested with CloneSelect Imager system and MTT assay**. The limits of agreement (bias ± 1.96 SD) are denoted with horizontal solid lines.

To compare the cytotoxicity results observed with the CloneSelect™ Imager system and the MTT assay, the agreement between the pEC_50 _values obtained using both methods can be calculated, using the so-called Bland-Altman plot [10]. For a comparison between the MTT assay and the SRB assay such a Bland-Altman plot was reported earlier [4], showing that the results obtained with the SRB assay displayed a good agreement with the MTT assay results. By plotting the difference between the pEC_50 _values determined by each of the methods against the mean of the two determinations it is possible to determine the agreement between both methods [10]. Therefore, to compare the two methods, a Bland-Altman analysis of the pEC_50 _values, obtained using the MTT assay and the CloneSelect™ Imager system, has been performed. For example, the Bland-Altman plot of cisplatin after 48 h of incubation in both the A2780 (Figure 3A) and the A2780R cells (Figure 3B) shows that there is a good agreement between the two variables, where all the data points are within the limits of agreement.

The limits of agreement are also calculated to give a quantitative analysis of the differences between both methods after 48 h and 72 h of incubation with cisplatin and oxaliplatin (Table 1). These results demonstrate that the pEC_50 _values obtained with the CloneSelect™ Imager system and the MTT assay are in close agreement. These observations clearly show that the CloneSelect Imager system is a very good alternative to the colorimetric assay to determine the antiproliferative activity of different drugs.

**Table 1:** Comparison of the pEC_50 _values obtained with the CloneSelect Imager system analysis and the MTT assay.

	Cell line/time	Mean difference	SD	Limits of agreement	
Compound				Mean-2SD	Mean+2SD
cisplatin	A2780/48 h	-0.64	0.24	-1.11	-0.17
	A2780R/48 h	0.12	0.08	-0.03	0.27
	A2780/72 h	-0.26	0.06	-0.36	-0.15
	A2780R/72 h	0.08	0.06	-0.04	0.20
oxaliplatin	A2780/48 h	-0.63	0.39	-1.40	0.13
	A2780R/48 h	-0.05	0.28	-0.60	0.51
	A2780/72 h	-0.18	0.22	-0.61	0.26
	A2780R/72 h	-0.11	0.07	-0.23	0.02

**Figure 4:**
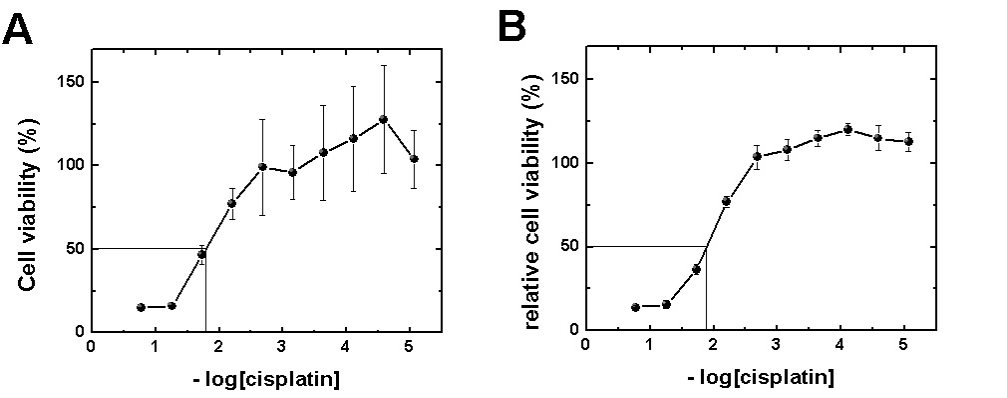
**Comparison of the dose response plot of cisplatin after 72 h in the A2780R cells**. Both plots display cell viability relative to non-treated cells *vs *-log [cisplatin]. (A) measure with the MTT assay and (B) measure with the CloneSelect Imager system. The pEC_50 _value is denoted at the crossing of the solid lines. The concentration of cisplatin used (8.47 nM, 25.4 nM, 76.2 nM, 0.229 μM, 0.686 μM, 2.06 μM, 6.17 μM, 18.5 μM, 55.6 μM, and 167 μM) plotted as -log C *vs *cell viability. The error bars indicate the accuracy and reproducibility.

In order to estimate the cytotoxic activity of the platinum compounds measured with the CloneSelect Imager, the data analysis using the non-treated cells as 100% viable cells at the end of the assay was performed. From independent measurements preformed using MTT and CSI it is noted that the MTT assay shows larger intra-experimental deviations than the CloneSelect Imager system (note the smaller error bars when CloneSelect Imager is used). For instance, the dose-respond plots for cisplatin incubation after 72 h in the A2780R cells (Figure 4A) show larger deviations within the data using the MTT assay than when using the CloneSelect Imager system (Figure 4B). The reproducibility is quite good when CSI is used, as shown by the small errors bars in the figure. However, larger deviations are observed for the MTT assay, as the number of viable cells increases and could account for often observed the poor linearity of the MTT assay with the cell number at high cell densities [14,15]. Similar results have been observed when the A2780 cells are incubated with cisplatin (data not shown).

### Cytotoxic activity in mice leukemia cells using CloneSelect™ Imager system

When using the MTT assay with non-adherent cells the MTT assay displays a huge disadvantage, since the culture medium has to be removed to quantify the formazan crystals at the end of the assay. Consequently, the last step in the protocol for the MTT assay results in the loss of crystals, and therefore, in large errors.

An improved colorimetric assay for non-adherent cells was introduced [16,17], using the substrate 5-(3-carboxymethoxyphenyl)-2-(4,5-dimethylthiazolyl)3-(4-sulfophenyl) tetrazolium (known as MTS), where the formazan product is soluble in the cell culture medium; however, unreliable results from the MTS assay in the L1210 cell line have been reported [18].

As a result of the difficulties of measuring cell cytotoxicity in non-adherent cells the CloneSelect Imager system was tested to determine if it was advantageous in this case. This study was undertaken using the known cytotoxic activity of cisplatin and oxaliplatin in the L1210/0 and the resistant counterpart L1210/2 cells. These cell lines are generally slightly attached to the bottom of the well, and careful handling of the microtiter plate allows accurate imaging with the CloneSelect Imager. The results for the different incubation times are summarized in Table 2.

**Table 2:** EC_50 _(μM) values obtained after 48 h and 72 h incubation with cisplatin and oxaliplatin on the suspension cultures L1210/0 and the L1210/2 cells obtained, using the CloneSelect Imager system

*compound*	*L1210/0* *48 h*	*L1210/2* *48 h*	RF*	*L1210/0* *72 h*	*L1210/2* *72 h*	RF*
Cisplatin	5.9	36.3	6.2	2.9	18.2	6.3
Oxaliplatin	3.8	5.6	1.5	2.0	1.3	0.7

*RF = resistance factor (EC_50 _(L1210/2)/EC_50_(L1210/0)

As expected, oxaliplatin displays a high cytotoxic activity against the L1210/0 cells [19], and it is able to overcome the resistance to cisplatin present in the L1210/2 cells, with no significant differences (*p *> 0.05) between the pEC_50 _values in the L1210/0 and in the L1210/2 cells for both incubation times. The MTT assay using the mice leukemia cells could not be performed in this case, because the last step in the MTT assay requires the discharge of the medium, generating experimental errors due to the loss of formazan crystals from the non-adherent cells, as mentioned above. This further illustrates the superiority of the CloneSelect Imager method.

## Conclusions

The most commonly used approach to quantify mammalian cell proliferation is the conversion of a tetrazolium salt (MTT) to the formazan crystals by cellular dehydrogenases [1]. Nevertheless, the MTT assay is time consuming when many samples have to be assayed. Moreover, the colorimetric assay kits are relatively expensive, and most important, they are destructive, not allowing measurements over time, using the same microtiter plate. New imaging methods have been introduced more recently, such as CloneSelect™ Imager. In this study this new system is compared with the widely used MTT assay, because - despite its disadvantages - the MTT assay is largely used in the screening of the antiproliferative effect of new compounds. A major advantage of the CloneSelect Imager system, compared to MTT assay, is that allows a direct screening of the antiproliferative activity of the drugs in only three minutes, with a direct overview from the cell confluence (%) after imaging in each well, not requiring additional steps to obtain these preliminary data. Additionally, it is possible to image the same plate as a function of time, based on a non-invasive methodology, saving in this way time and costs.

In summary, the CloneSelect™ Imager system has been compared with the MTT assay, and it displays lower intra experimental deviations in the dose response curves, with high reproducibility, and showing that the CloneSelect Imager system is a much preferred alternative to determine the antiproliferative activity of different drugs and possibly more accurate.

## Methods

### Platinum compounds

The platinum compounds cisplatin and oxaliplatin were purchased from Aldrich. Aliquots of a 2 mM stock solution of cisplatin and oxaliplatin in 40 mM NaCl were stored in dark at -20°C, defrosted and diluted with cell culture medium to the desired concentration before use.

### Cell lines, culture conditions

The human ovarian carcinoma cell line A2780 and its cisplatin-resistant counterpart A2780R, and the cisplatin-sensitive mouse leukemia L1210/0 and its cisplatin-resistance counterpart L1210/2 were grown in suspension at 37°C in a 7% CO_2 _atmosphere, and were maintained in a continuous logarithmic culture in Dulbecco´s modified Eagle´s Medium (DMEM) (Gibco BRL TM, Invitrogen Corporation, The Netherlands) supplemented with 10% heat-inactivated fetal calf serum (Hyclone, Perbio Science, The Netherlands), penicillinG Sodium (100 units/ml: Dufecha, Biochemie BV, The Netherlands), streptomycin (100 μg/ml: Dufecha, Biochemie BV, The Netherlands) and Glutamax 100× (Gibco BRL TM, The Netherlands).

### Cytotoxicity assay

Microtiter plates with ninety six wells were used for the antiproliferative measurements of cisplatin and oxaliplatin. Cultures in the well plates were measured at different time points, and returned to the incubator. The number of seeded cells was adjusted according to the incubation time with the drugs. On day one, 10,000 cell/well for 24 h incubation assay were seeded, while 5,000 and 3,000 cells/well were seeded for 48 h and 72 h incubation assay, respectively. The plates were pre-incubated for 24 h at 37°C, 7% CO_2 _to allow the human ovarian carcinoma cells to adhere. In case of the mice leukemia cells, the plates were also kept for 24 h before the addition of the platinum compounds. The L1210/0 and the L1210/2 cells are in suspension or slightly attached. On day two, the plates were monitored with the CloneSelect™ Imager system (Genetix, UK) before the addition of the antiproliferative agents (cisplatin and oxaliplatin) and returning to the incubator. A threefold dilution series of ten concentrations of the platinum drugs was made, starting with a 2 mM stock solution of the compounds. For each concentration 50 μL of compound was added to the medium in triplicate. All time-point experiments were performed in parallel and were repeated five times, independently to verify how reproducible the experiments are. To obtain the antiproliferative data with CloneSelect Imager and MTT from the same plate at the same time point the treated microtiter plates were first monitored using CloneSelect Imager system and subsequently 50 μL of a MTT solution (5 mg/ml in PBS buffer) were added to each well, and the plate kept at 37°C for 3 h to allow formation of the formazan crystals [1]. The solution was then carefully removed and the blue formazan crystals were dissolved in 100 μL DMSO, and the absorbance was read at 590 nm using an automatic microplate reader (Labsystems Multiskan MS) as previously described [20,21]. In the case of the mice leukemia cells, the plates were imaged with the CSI system. The data obtained with CloneSelect Imager assay and with MTT assay were analyzed and the pEC_50 _values (EC_50 _is the drug concentration that produces 50% of the maximum response) were determined with the GraphPad Prism™ analysis software package (GraphPad Software, San Diego, USA) using non-linear regression (sigmoidal dose response, variable slope).

### Statistical analysis

The comparison of the antiproliferative activity determined by the two methods (CloneSelect Imager and MTT) was performed using the Bland-Altman plot [10]. In the cytotoxic activity the Student's t-test was performed for statistical comparison. A *p*-value < 0.05 was considered as statistically significant.

## Abbreviations

MTT: 3-(4,5-dimethylthiazol-2yl)-2,5-diphenyl-2H-tetrazolium bromide; EC_50 _values: drug concentration that produces 50% of the maximum possible response, pEC_50 _values = -log EC_50_

## Competing interests

Julian F Burke declares competing financial interests. The other authors declare they have no competing interests.

## Authors' contributions

HD performed the experiments in the L1210/0 and the L1210/2 cells. CB participated in the study design, and contributed to drafting the manuscript. JB, JR and JF participated in discussion of the results and contributed to drafting and finalising the manuscript. PM designed the experiment, performed data analysis and wrote the article. All authors read and approved the final manuscript.

## Supplementary Material

Additional file 1 **Figure S1**. Cell confluence (%) *vs *incubation time with cisplatin in the A2780 cells. Click here for file

Additional file 2 **Figure S2**. Cell confluence (%) *vs *incubation time with cisplatin in the A2780 cells. Click here for file

Additional file 3 **Figure S3**. Cell confluence (%) of the A2780 cells treated with cisplatin or oxaliplatin after 72 h of incubation. Click here for file
