# Supplementary material for: Accurate non-invasive image-based cytotoxicity assays for cultured cells
Source: BMC Biotechnol. 2010 Jun 17;10:43. doi: 10.1186/1472-6750-10-43 (PMC2906423; doi:10.1186/1472-6750-10-43)

Additional file 1, marques

**Figure S1**. An example of the CSI plot of the cell confluence (%) *vs* incubation time with cisplatin in the A2780 cells. Text has been added for clarity, and the negative control is highlighted with a red line. The compounds are added to the cells in ten different concentrations (from lowest to highest: 8.47 nM, 25.4 nM, 76.2 nM, 0.229 µM, 0.686 µM, 2.06 µM, 6.17 µM, 18.5 µM, 55.6 µM, and 167 µM).


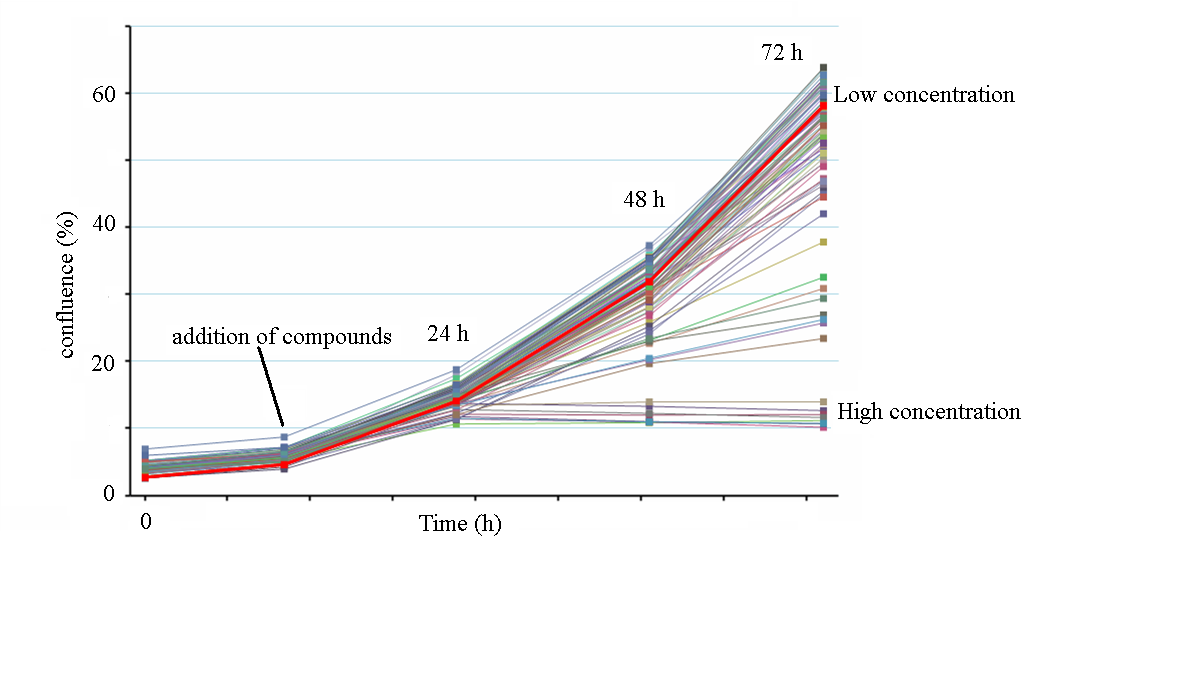

Supplement: Additional file 1 — Figure S1. Cell confluence (%) vs incubation time with cisplatin in the A2780 cells. [file 1472-6750-10-43-S1.DOC]
