# Supplementary material for: Accurate non-invasive image-based cytotoxicity assays for cultured cells
Source: BMC Biotechnol. 2010 Jun 17;10:43. doi: 10.1186/1472-6750-10-43 (PMC2906423; doi:10.1186/1472-6750-10-43)
Supplement: Additional file 2 — Figure S2. Cell confluence (%) vs incubation time with cisplatin in the A2780 cells. [file 1472-6750-10-43-S2.DOC]

Addtional file 2, Marques

**Figure S2**. Cell confluence (%) *vs* incubation time with cisplatin in the A2780 cells; only results of six wells are plotted for clarity.
