# Supplementary material for: Accurate non-invasive image-based cytotoxicity assays for cultured cells
Source: BMC Biotechnol. 2010 Jun 17;10:43. doi: 10.1186/1472-6750-10-43 (PMC2906423; doi:10.1186/1472-6750-10-43)

Additional file 3, Marques

**Figure S3.** Cell confluence (in %) of the A2780 cells treated with cisplatin or oxaliplatin after 72 h of incubation. Column 1 and 2 represent the negative controls. From column 3 till column 12 ten different concentrations are used (from the left to the right: 8.47 nM, 25.4 nM, 76.2 nM, 0.229 µM, 0.686 µM, 2.06 µM, 6.17 µM, 18.5 µM, 55.6 µM, and 167 µM).


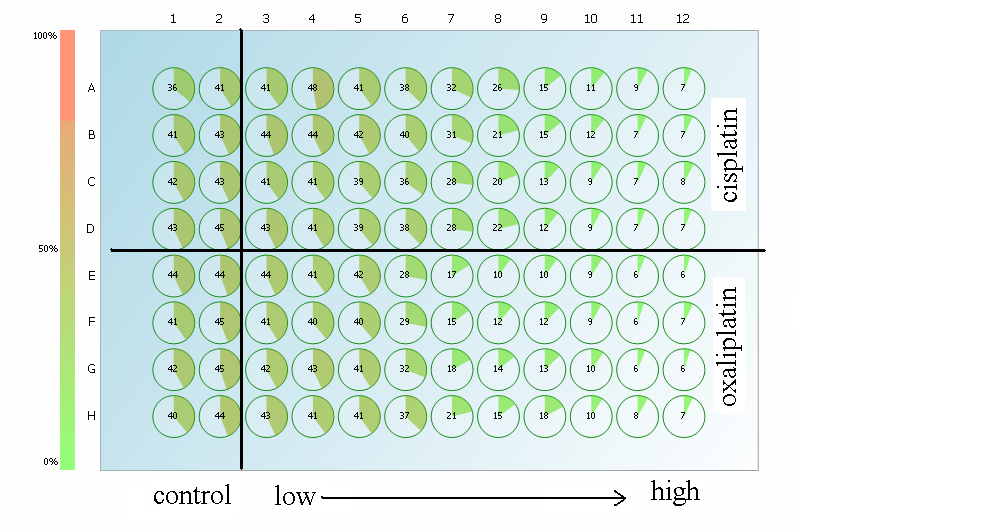

Supplement: Additional file 3 — Figure S3. Cell confluence (%) of the A2780 cells treated with cisplatin or oxaliplatin after 72 h of incubation. [file 1472-6750-10-43-S3.DOC]
